# Traumatic brain injury alters dendritic cell differentiation and distribution in lymphoid and non-lymphoid organs

**DOI:** 10.1186/s12974-022-02609-5

**Published:** 2022-10-01

**Authors:** Orest Tsymbalyuk, Volodymyr Gerzanich, J. Marc Simard, Chozha Vendan Rathinam

**Affiliations:** 1 Department of Neurosurgery, University of Maryland School of Medicine, MD Baltimore, USA; 2 Institute of Human Virology, University of Maryland School of Medicine, 725 West Lombard Street, Baltimore, MD 21201 USA; 3 Research Service, Veterans Affairs Maryland Health Care System, MD Baltimore, USA; 4 Department of Pathology, University of Maryland School of Medicine, MD Baltimore, USA; 5 Department of Physiology, University of Maryland School of Medicine, MD Baltimore, USA; 6 Center for Stem Cell and Regenerative Medicine, University of Maryland School of Medicine, MD 21201 Baltimore, USA

**Keywords:** Traumatic brain injury, Dendritic cells, Immune system, Dendritic cell progenitors, Reactive oxygen species

## Abstract

**Background:**

Pathophysiological consequences of traumatic brain injury (TBI) mediated secondary injury remain incompletely understood. In particular, the impact of TBI on the differentiation and maintenance of dendritic cells (DCs), which are regarded as the most professional antigen presenting cells of the immune system, remains completely unknown. Here, we report that DC-differentiation, maintenance and functions are altered on day 3 and day 7 after TBI.

**Methods:**

Long bones, spleen, peripheral lymph nodes (pLNs), mesenteric lymph nodes (mLNs), liver, lungs, skin and blood were collected from mice with either moderate-level cortical impact (CCI) or sham on day 1, day 3 or day 7 after TBI. Bone marrow cells were isolated from the tibias and femurs of hind limb through flushing. Tissues were digested with Collagenase-D and DNase I. Skin biopsies were digested in the presence of liberase + DNase I. Single cell suspensions were made, red blood cells were lysed with Ammonium chloride (Stem Cell Technology) and subsequently filtered using a 70 μM nylon mesh. DC subsets of the tissues and DC progenitors of the BM were identified through 10-color flow cytometry-based immunophenotyping studies. Intracellular reactive oxygen species (ROS) were identified through H2DCFDA staining.

**Results:**

Our studies identify that; (1) frequencies and absolute numbers of DCs in the spleen and BM are altered on day 3 and day 7 after TBI; (2) surface expression of key molecules involved in antigen presentation of DCs were affected on day 3 and day 7 after TBI; (3) distribution and functions of tissue-specific DC subsets of both circulatory and lymphatic systems were imbalanced following TBI; (4) early differentiation program of DCs, especially the commitment of hematopoietic stem cells to common DC progenitors (CDPs), were deregulated after TBI; and (5) intracellular ROS levels were reduced in DC progenitors and differentiated DCs on day 3 and day 7 after TBI.

**Conclusions:**

Our data demonstrate, for the first time, that TBI affects the distribution pattern of DCs and induces an imbalance among DC subsets in both lymphoid and non-lymphoid organs. In addition, the current study demonstrates that TBI results in reduced levels of ROS in DCs on day 3 and day 7 after TBI, which may explain altered DC differentiation paradigm following TBI. A deeper understanding on the molecular mechanisms that contribute to DC defects following TBI would be essential and beneficial in treating infections in patients with acute central nervous system (CNS) injuries, such as TBI, stroke and spinal cord injury.

## Background

Traumatic brain injury (TBI) is one of the major causes of morbidity and disability worldwide [1, 2]. Approximately, 1.7 million people per year are afflicted by TBI and contributes to 30% of all injury-related deaths with an annual cost of ~ $60 billion within the United States [1, 3–6]. In addition to causing severe trauma in the brain tissue, TBI initiates a cascade of cellular, molecular and biochemical events that are referred to as “secondary injury” [7]. Indeed, clinical outcome of TBI is determined by the nature and severity of both primary and secondary injuries. While the clinical significance of tightly controlled neuroinflammation has begun to unfold, the pathophysiological consequences of TBI mediated secondary injury remain incompletely understood.

Dendritic cells (DCs) are the most potent antigen presenting cells and function as the sentinels of the immune system. DCs initiate and shape both innate and adaptive immune responses [8, 9]. In particular, DCs are essential for the initiation of protective T- and B-cell responses and, thus constitute a frontline defense against invading pathogens [10–12]. Indeed, impaired DC differentiation and functions ultimately results in severe autoimmune deficiencies and enhanced susceptibility to viral, bacterial and fungal infections in mice and humans [13–15]. DCs are present in most tissues of the body and can be broadly divided into three major groups; (1) conventional or classical DCs (cDCs), which are further subdivided into cDC1 and cDC2 subsets, (2) plasmacytoid DCs (pDCs) and (3) monocyte-derived DCs (moDCs) [11, 16, 17]. cDCs patrol the local environment, actively engulf and sample foreign antigens, migrate to the T-cell zones of the draining lymph nodes, and present antigenic peptides to antigen inexperienced/naïve T cells. pDCs are, particularly, essential for the production of type I interferons and the establishment of anti-viral immunity. On the other hand, moDCs are routinely generated in vitro from monocytes in presence of granulocyte–macrophage colony stimulating factor (GM-CSF, CSF-2) and interleukin4 (IL4) and are often utilized under clinical settings to perform immunotherapies against cancer [17].

Earlier studies unequivocally established the impact of TBI on peripheral immune cells, including both innate (granulocytes and macrophages) and adaptive (T-, B-, and NK-cells) immune system [18–21]. A series of recent studies highlighted the significance of DC-mediated functions during CNS injuries, including TBI [22], middle cerebral artery occlusion and cerebral ischemia [23]. However, physiological consequences of TBI on the development of DCs remain totally unknown. Our previous studies [24–27], as well as of others, established that sustained and chronic inflammation impairs early differentiation pathways in HSCs. In particular, our studies indicated that the Flt3^+^ hematopoietic progenitors are more sensitive to acute and chronic inflammation. Based on the key roles of Flt3/Flt3L signaling axis in the differentiation and maintenance of DCs, we hypothesized that TBI-induced inflammation affects differentiation and maintenance of peripheral DCs.

In the present study, we induced experimental TBI in mice and studied its impact on DC maintenance. Our data identified that TBI affects the distribution pattern of DCs and induces an imbalance among DC subsets in both lymphoid and non-lymphoid organs. In addition, the current study demonstrates that TBI results in reduced levels of reactive oxygen species (ROS) in DCs on day 3 and day 7 after TBI, which may explain altered DC differentiation paradigm following TBI.

## Methods

### Mice and controlled cortical impact injury

**Fig. 1:**
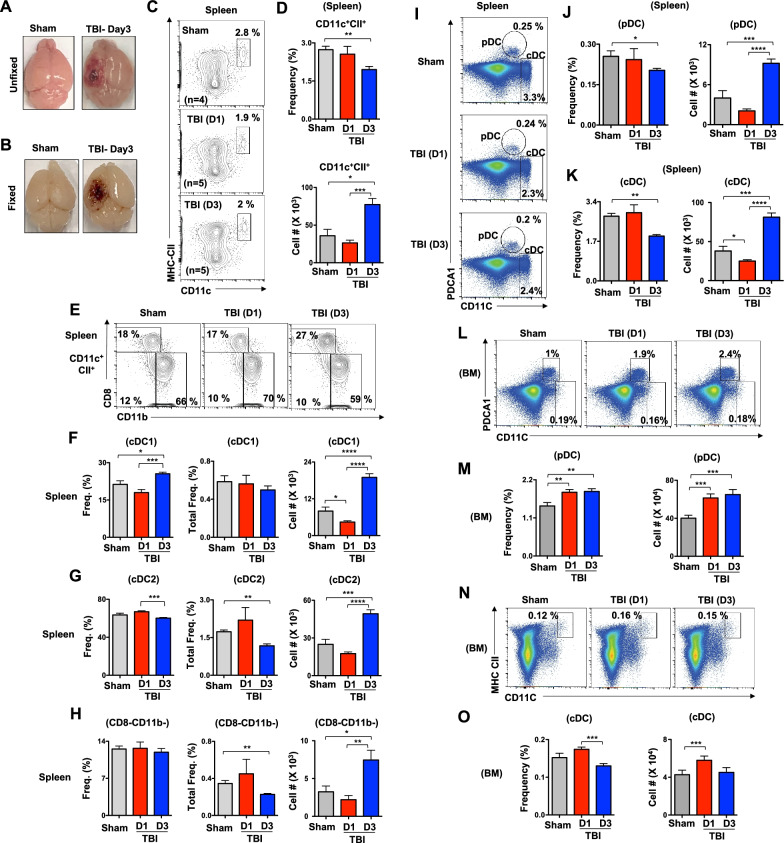
TBI leads to altered numbers and distribution of DC subsets at the early phase. **A** Images of unfixed brains from sham and d3-TBI mice. **B** Images of formalin fixed brains from sham and d3-TBI mice. **C** FACS plots indicating frequencies of CD11c^+^MHC-Class II^high^ cDCs in the spleen of Sham, d1-TBI and d3-TBI mice. Data are representative of two independent experiments. **D** Cumulative frequencies (top) and absolute numbers (bottom) of cDCs in the spleen of Sham (*n* = 4), d1-TBI (*n* = 5) and d3-TBI (*n* = 5) mice. Data are representative of two independent experiments. **E** FACS plots indicating frequencies of CD8^+^CD11b^−^cDC1, CD8^−^CD11b^+^cDC2 and CD8^−^CD11b^−^ immature cDCs within pre-gated CD11c^+^MHC-Class II^high^ cDCs in the spleen of Sham (*n* = 4), d1-TBI (*n* = 5) and d3-TBI (*n* = 5) mice. Data are representative of two independent experiments. **F**–**H** Relative frequencies (left), overall frequencies (middle) and absolute numbers (right) of cDC1 (**F**), cDC2 (**G**) and immature cDCs (**H**) subsets in the spleen of Sham (*n* = 4), d1-TBI (*n* = 5) and d3-TBI (*n* = 5) mice. Data are representative of two independent experiments. **I** FACS plots indicating frequencies of PDCA1^+^CD11c^int^ pDCs and PDCA1^−^CD11c^high^ cDCs in the spleen of Sham (*n* = 4), d1-TBI (*n* = 5) and d3-TBI (*n* = 5) mice. Data are representative of two independent experiments. **J**, **K** Overall frequencies (left) and absolute numbers (right) of pDCs (**J**) and cDCs (**K**) in the spleen of Sham (*n* = 4), d1-TBI (*n* = 5) and d3-TBI (*n* = 5) mice. Data are representative of two independent experiments. **L** FACS plots indicating frequencies of PDCA1^+^CD11c^int^ pDCs and PDCA1^−^CD11c^high^ cDCs in the BM of Sham (*n* = 4), d1-TBI (*n* = 5) and d3-TBI (*n* = 5) mice. Data are representative of two independent experiments. **M** Overall frequencies (left) and absolute numbers (right) of pDCs in the BM of Sham (*n* = 4), d1-TBI (*n* = 5) and d3-TBI (*n* = 5) mice. Data are representative of two independent experiments. **N** FACS plots indicating frequencies of CD11c^+^MHC-ClassII^high^ cDCs in the BM of Sham (*n* = 4), d1-TBI (*n* = 5) and d3-TBI (*n* = 5) mice. Data are representative of two independent experiments. **O** Overall frequencies (left) and absolute numbers (right) of cDCs in the BM of Sham (*n* = 4), d1-TBI (*n* = 5) and d3-TBI (*n* = 5) mice. Data are representative of two independent experiments. All data represent mean ± SEM. Mann–Whitney non-parametric tests were used to assess statistical significance (*P < 0.05, **P < 0.01, *** *P* < 0.001, **** *P* < 0.0001)

C57BL/6 mice were purchased from the Jackson Laboratory. 8–12-week mice were used in this study. After being anesthetized with isoflurane, the subject mice received controlled cortical impact (CCI) using a custom microprocessor-controlled and compressed air driven pneumatic impactor. Briefly, a 10-mm midline incision was made over the skull, the skin and fascia were retracted, and a 4-mm craniotomy was made on the central aspect of the left parietal bone of mice under surgical anesthesia. A moderate injury was induced by a 3.5-mm diameter tip with impact velocity of 6 m/s and a deformation depth of 2 mm (Fig. 1A, B). In sham mice, same procedure was performed except for the impact. The number of mice in each study is indicated in the figure legends. All surgical procedures and animal experiments were performed under protocols approved by the University of Maryland School of Medicine Institutional Animal Care and Use Committee (IACUC). The surgical procedures were performed by the same investigator using the same equipment.

### Cell preparation

Spleen, lymph nodes (LNs), liver, lungs were digested with Collagenase-D (0.7 mg/mL) and DNase I (100U/ mL) in RPMI for 45 min at 37 °C. For skin DC isolation, mouse ears were cut into pieces, digested in the presence of 250 μg/mL liberase + DNase I (100U/ mL) in HBSS + 5% FCS for 90 min at 37 °C. Cells were vortexed vigorously and passed through a 19-gauge needle to obtain a single cell suspension. Bone marrow cells were isolated from the tibias and femurs by inserting a 23-gauge needle/ 1 mL syringe to the bone cavities and flushed with PBS 2%FCS until the bones become pale. Single cell suspensions were made through rigorous pipetting.

Red blood cells were lysed with Ammonium chloride (Stem Cell Technology) and subsequently filtered using a 70 μM nylon mesh. Bone marrow cells were then counted with a hemacytometer and trypan blue (Amresco) negative cells were counted as live cells.

### Flow cytometry

Cells were analyzed by flow cytometry with Attune Nxt (Thermofisher) and FlowJo software (Tree Star). The following monoclonal antibodies were used: anti-CD34 (RAM34), anti-CD48 (HM48-1), anti-CD117 (2B8), anti-Flt3 (A2F10.1), anti-Sca-1 (D7), anti-B220 (RA3-6B2), anti-CD19 (1D3), anti-CD3 (145-2C11), anti-CD4 (GK1.5), anti-CD8 (53-6.7), anti-CD11b (M1/70), anti-Gr-1 (RB6-8C5), anti-Ter119 (TER119), anti-CD11c (N418), anti-CD8(53-6.7), anti-PDCA1 (129C1), anti-CD24 (M1/69), anti-SIRPa (P84), anti-CD103 (2E7), anti-CD45 (S18009F), anti-CD207 (4C7), anti-CD115 (AFS98), anti-LY6C (HK1.4), anti-SiglecH (551), anti-CLEC9A (7H11), anti-CD80 (16-10A1), anti-CD86 (GL-1), anti-CD40 (3/23), anti-H2K^b^ (AF6-88.5), anti-IA/IE (M5/114.15.2) and anti-BrdU (3D4) from Biolegend. Cells incubated with biotinylated monoclonal antibodies were incubated with fluorochrome-conjugated streptavidin–peridinin chlorophyll protein–cyanine 5.5 (551419; BD), streptavidin–allophycocyanin-Cy7 (554063; BD), streptavidin-super bright 650 (Biolegend). In all the FACS plots, indicated are the percentages (%) of the gated fraction. Data were acquired on a Attune Nxt Acoustic focusing cytometer using Attune software (life technologies) and analyzed using FlowJo (Treestar Inc).

### Measuring ROS levels

Single cell suspensions were stained with cell surface markers and then incubated with 2 mM CM-H_2_DCFDA (Life Technologies C6827) in pre-warmed HBSS at 37 °C for 15 min. The cells were washed in ice-cold PBS, pelleted and resuspended in ice-cold PBS 2% FCS.

### Statistics

Data represent mean and s.e.m. Significance was evaluated between 2 individual samples using Student unpaired *t *tests. Comparisons within each surgery group were analyzed using two-way ANOVA with multiple comparisons test. For non-parametric data, Mann–Whitney test was used (*P < 0.05, **P < 0.01, *** < 0.001, **** < 0.0001).

## Results

### Augmented DC pool size in the spleen and bone marrow at the acute phase of TBI

To study if TBI causes alterations in DC compartment, we enumerated the frequencies of DCs in the spleen and BM. Flow cytometry-based immunophenotyping studies indicated reduced relative frequencies, but increased absolute numbers, of CD11c^+^CII^+^ cDCs in the spleen after 3 days (d3) of TBI (Fig. 1C, D). Further fractionation of CD11c^+^CII^+^ DCs into CD8^+^CD11b^−^ (cDC1) and CD8^−^CD11b^+^ (cDC2) subsets [11, 16, 17] subsets revealed; normal relative numbers, but reduced absolute numbers of cDC1 after 1 day (d1) of TBI (Fig. 1E, F); an increase in relative frequencies, normal total frequencies and increased absolute numbers of cDC1 subset after d3 of TBI (Fig. 1E, F); reduced overall frequencies, but increased absolute numbers of cDC2 after d3 of TBI (Fig. 1E, G); and normal relative frequencies, reduced overall frequencies and increased absolute numbers of CD8–CD11b–CD11c^+^CII^+^ immature cDCs in the spleen after d3 of TBI (Fig. 1E–H). Further analysis indicated reduced relative numbers, but increased absolute numbers, of PDCA1^+^CD11c^int^ pDCs (Fig. 1I, J) and PDCA1^−^CD11c^high^ cDCs (Fig. 1I, K) in the spleen after d3 of TBI. Interestingly, normal relative frequencies, but reduced absolute numbers, of PDCA1^−^CD11c^high^ cDCs was observed in the spleen after d1 of TBI (Fig. 1I, K). Next, we determined the frequencies of DCs in the BM and analysis revealed an increase in both relative frequencies and absolute numbers of PDCA1^+^CD11c^int^ pDCs in the BM after d1 and d3 of TBI (Fig. 1L, M). On the other hand, frequencies of CD11c^+^CII^+^ cDCs were reduced, even though their absolute numbers were normal, in the BM after d3 of TBI (Fig. 1N, O). Finally, absolute numbers of CD11c^+^CII^+^ cDCs were increased, while the relative numbers were normal, in the BM after d1 of TBI (Fig. 1N, O). Overall, these data indicate that the frequencies and absolute numbers of DCs in the spleen and BM are altered at the acute phase of TBI.

### Altered DC subsets in the spleen and BM at the sub-chronic phase of TBI

**Fig. 2:**
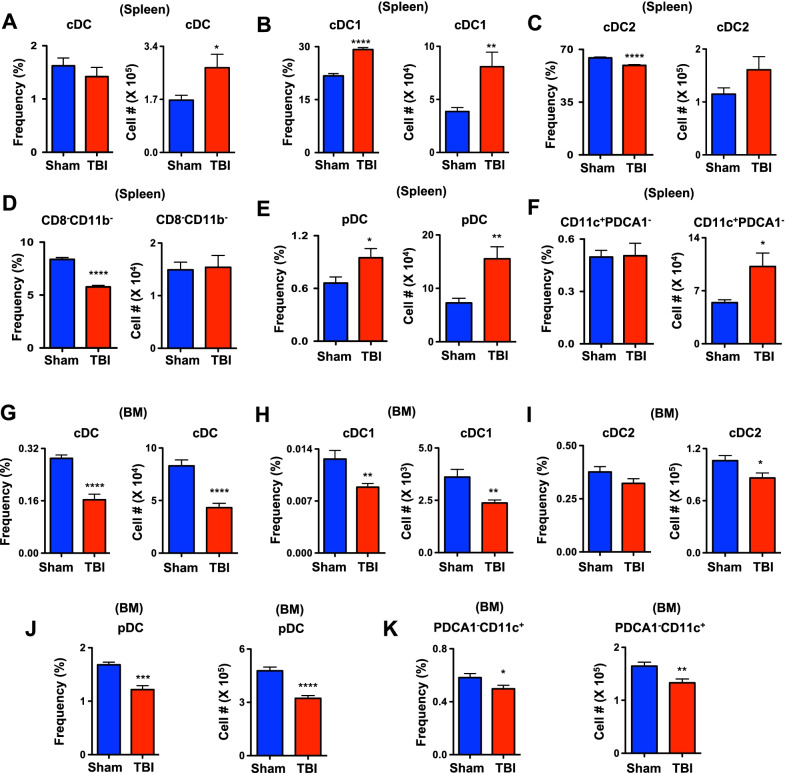
TBI causes changes in DC pool and functions at the sub-chronic phase. **A** Relative frequencies (left) and absolute numbers (right) of cDCs in the spleen of Sham (*n* = 4) and d7-TBI (*n* = 5) mice. Data are representative of two independent experiments. **B** Relative frequencies (left) and absolute numbers (right) of cDC1 subset in the spleen of Sham (*n* = 4) and d7-TBI (*n* = 5) mice. Data are representative of two independent experiments. **C** Relative frequencies (left) and absolute numbers (right) of cDC2 subset in the spleen of Sham (*n* = 4) and d7-TBI (*n* = 5) mice. Data are representative of two independent experiments. **D** Relative frequencies (left) and absolute numbers (right) of immature cDC subset in the spleen of Sham (*n* = 4) and d7-TBI (*n* = 5) mice. Data are representative of two independent experiments. **E** Relative frequencies (left) and absolute numbers (right) of pDC subset in the spleen of Sham (*n* = 4) and d7-TBI (*n* = 5) mice. Data are representative of two independent experiments. **F** Relative frequencies (left) and absolute numbers (right) of PDCA1^−^CD11c^high^ cDC subset in the spleen of Sham (*n* = 4) and d7-TBI (*n* = 5) mice. Data are representative of two independent experiments. **G** Relative frequencies (left) and absolute numbers (right) of cDCs in the BM of Sham (*n* = 4) and d7-TBI (*n* = 5) mice. Data are representative of two independent experiments. **H** Relative frequencies (left) and absolute numbers (right) of cDC1 subset in the BM of Sham (*n* = 4) and d7-TBI (*n* = 5) mice. Data are representative of two independent experiments. **I** Relative frequencies (left) and absolute numbers (right) of cDC2 subset in the BM of Sham (*n* = 4) and d7-TBI (*n* = 5) mice. Data are representative of two independent experiments. **J** Relative frequencies (left) and absolute numbers (right) of pDC subset in the BM of Sham (*n* = 4) and d7-TBI (*n* = 5) mice. Data are representative of two independent experiments. **K** Relative frequencies (left) and absolute numbers (right) of PDCA1^−^CD11c^high^ cDC subset in the BM of Sham (*n* = 4) and d7-TBI (*n* = 5) mice. Data are representative of two independent experiments. All data represent mean ± SEM. Two-tailed student’s *t* tests were used to assess statistical significance (*P < 0.05, **P < 0.01, *** *P* < 0.001, **** *P* < 0.0001)

To investigate the impact of TBI on DC subsets at the sub-chronic phase, we performed immunophenotyping studies after 7 days (d7) of TBI. Our studies indicated normal frequencies, but increased absolute numbers, of CD11c^+^CII^+^ cDCs in the spleen of d7 TBI mice (Fig. 2A). Further analysis of splenic cDC fraction of d7 TBI mice indicated; increased frequencies and absolute numbers of cDC1 subset (Fig. 2B); reduced frequencies, but normal absolute numbers, of cDC2 subset (Fig. 2C); and reduced frequencies, but normal absolute numbers, of immature cDC subset (Fig. 2D). Analysis of pDC compartment indicated increased frequencies and absolute numbers in spleen of d7 TBI mice (Fig. 2E). Consistent with data shown in Fig. 2A, frequencies of PDCA1^−^CD11c^high^ were normal, but their absolute numbers were augmented in the spleen of d7 TBI mice (Fig. 2F).

On the other hand, analysis of DC subsets in the BM of d7 TBI mice revealed; a remarkable decrease in both frequencies and absolute numbers of CD11c^+^CII^+^ cDCs (Fig. 2G); reduced frequencies and absolute numbers of cDC1 (Fig. 2H); non-significant reduction in relative frequencies, but a reduction in absolute numbers, of cDC2 (Fig. 2I); decreased relative frequencies and absolute numbers of pDCs (Fig. 2J); and reduced frequencies and absolute numbers of PDCA1^−^CD11c^high^ cDCs (Fig. 2K). In essence, these data specify that sub-chronic phase of TBI induces an imbalance in the distribution of cDC and pDC subsets in the spleen and BM.

### TBI alters expression levels of surface markers involved in DC functions

**Fig. 3:**
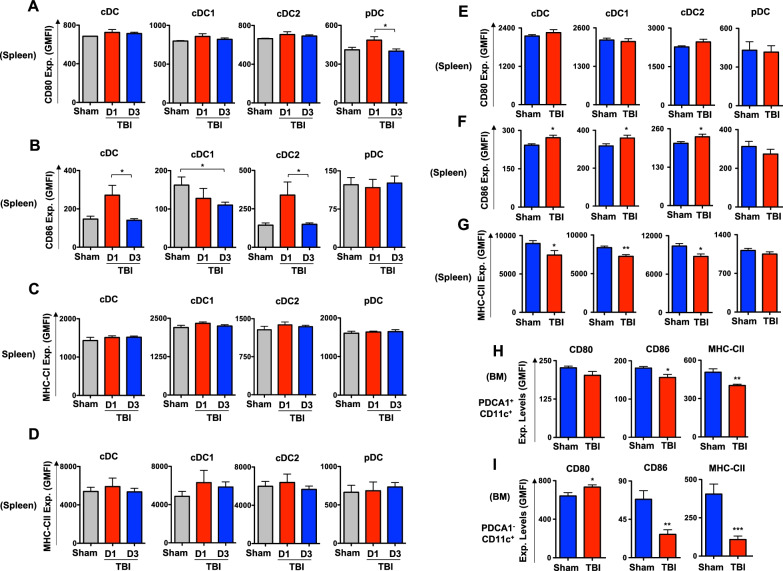
TBI alters expression of expression levels of surface molecules involved in DC functions. **A**–**D** Surface expression levels of CD80 (**A**), CD86 (**B**), MHC-CI (**C**) and MHC-CII (**D**) in total cDC, cDC1, cDC2 and pDC subsets of spleen from Sham (*n* = 4), d1- (*n* = 5) and d3- (*n* = 5) TBI mice. Shown were Geo-Mean Fluorescence Intensities (GMFI). **E**–**G** Surface expression levels of CD80 (**E**), CD86 (**F**) and MHC-CII (**G**) in total cDC, cDC1, cDC2 and pDC subsets of spleen from Sham (*n* = 4) and d7-TBI (*n* = 5) mice. Shown were GMFI. **H**, **I** Surface expression levels of CD80, CD86 and MHC-CII in PDCA1^+^CD11c^+^ pDC (**H**) and PDCA1^−^CD11c^+^ cDC (**I**) subsets of the BM from Sham (*n* = 4), d1- (*n* = 5) and d3- (*n* = 5) TBI mice. Shown were GMFI. All data represent mean ± SEM. Mann–Whitney non-parametric tests and two-tailed student’s *t* tests were used to assess statistical significance (*P < 0.05, **P < 0.01, ***P < 0.001, ****P < 0.0001)

To test if expression of surface antigens essential for DC functions is affected by TBI, we performed detailed immunophenotyping studies. Analysis indicated CD80 expression was normal in total DCs, cDC1 and cDC2 subsets of the spleen after d1 and d3 of TBI (Fig. 3A). However, CD80 expression was modestly reduced in splenic pDCs after d3, when compared with d1, of TBI (Fig. 3A). Expression of CD86 was increased on total DCs and cDC2 subset on d1, when compared with that of d3 after TBI (Fig. 3B). Interestingly, CD86 expression levels in cDC1 subset were reduced on d3 after TBI and in pDCs remain unchanged after TBI (Fig. 3B). More importantly, expression levels of MHC class I (Fig. 3C) and MHC class II (Fig. 3D) were normal in total DCs, cDC1, cDC2 and pDCs of spleen after d1 and d3 of TBI.

Next, we assessed if DC associated surface markers are altered at the sub-chronic phase of TBI. Analysis on expression levels of surface antigen in splenic DC subsets of d7 TBI mice revealed; normal expression levels of CD80 in cDC, cDC1, cDC2 and pDC subsets (Fig. 3E); increased expression levels of CD86 in total cDC, cDC1 and cDC2 subsets, whereas normal levels of CD86 in pDCs (Fig. 3F); and reduced MHC-CII expression in total cDC, cDC1 and cDC2 subsets, whereas normal levels of MHC-CII in pDCs (Fig. 3G). Immunophenotyping studies on BM DC subsets of d7 TBI mice indicated decreased MHC-CII and CD86 expression, whereas normal CD80 expression, in pDC subset (Fig. 3H) and striking reduction of MHC-CII and CD86 expression, whereas increased levels of CD80 expression, in PDCA1^−^CD11C^+^ cDC fraction (Fig. 3I). Overall, these data indicate that TBI affects surface expression of molecules involved in antigen presentation of DCs on day 3 and day 7 after TBI.

### TBI causes an imbalance in DCs subsets within the circulatory and lymphatic systems

**Fig. 4:**
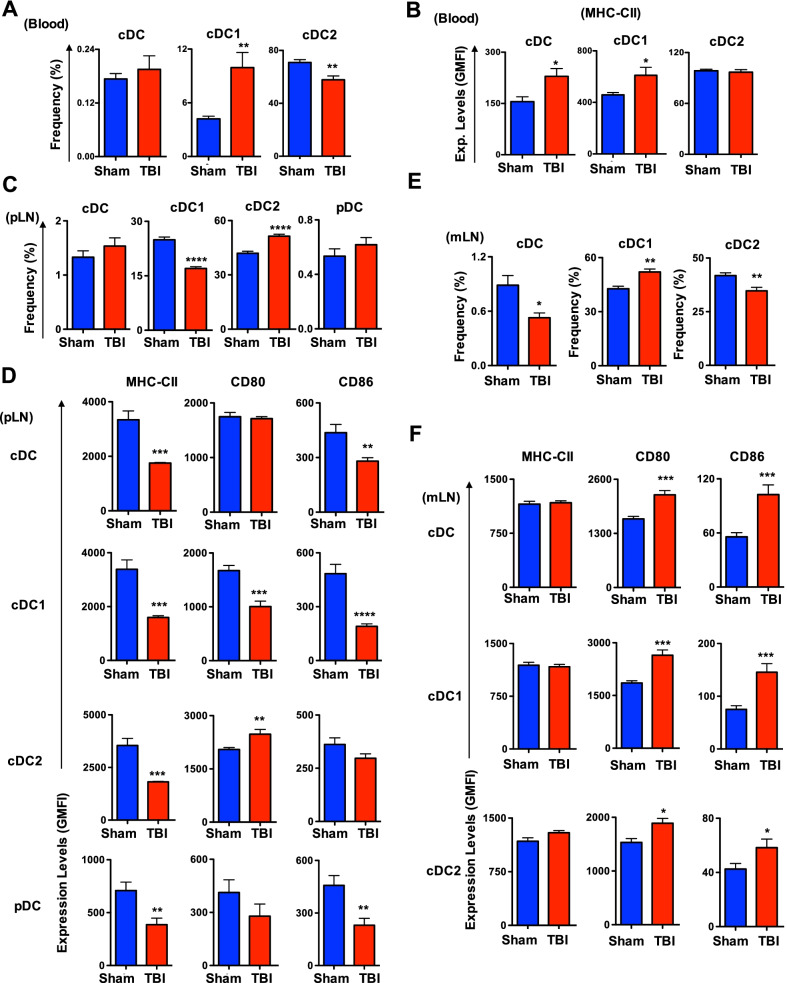
TBI results in an imbalance among DC subsets of the circulatory and lymphatic systems. **A** Relative frequencies of total cDC, cDC1 and cDC2 subsets in the peripheral blood from Sham (*n* = 4) and d7-TBI (*n* = 5) mice. Data are representative of three independent experiments. **B** Surface expression levels of MHC-CII in total cDC, cDC1 and cDC2 subsets of peripheral blood from Sham (*n* = 4) and d7-TBI (*n* = 5) mice. Shown were GMFI. Data are representative of three independent experiments. **C** Relative frequencies of total cDC, cDC1, cDC2 pDC subsets in the peripheral lymph nodes from Sham (*n* = 4) and d7-TBI (*n* = 5) mice. Data are representative of two independent experiments. **D** Surface expression levels of MHC-CII, CD80 and CD86 in total cDC, cDC1, cDC2 and pDC subsets of peripheral lymph nodes from Sham (*n* = 4) and d7-TBI (*n* = 5) mice. Shown were GMFI. Data are representative of two independent experiments. **E** Relative frequencies of total cDC, cDC1 and cDC2 subsets in the mesenteric lymph nodes from Sham (*n* = 4) and d7-TBI (*n* = 5) mice. Data are representative of two independent experiments. **F** Surface expression levels of MHC-CII, CD80 and CD86 in total cDC, cDC1, cDC2 and pDC subsets of mesenteric lymph nodes from Sham (*n* = 4) and d7-TBI (*n* = 5) mice. Shown were GMFI. Data are representative of two independent experiments. All data represent mean ± SEM. Two-tailed student’s *t* tests were used to assess statistical significance (*P < 0.05, **P < 0.01, ***P < 0.001, ****P < 0.0001)

Under steady state conditions DCs are constantly circulated throughout the body to perform immune surveillance and distributed to different organs through sophisticated networks of the blood and lymphatic systems [8–12]. To study if TBI has an impact on circulating DCs, we determined their frequencies in the blood and lymph nodes. Analysis of peripheral blood indicated normal frequencies of total cDCs (Fig. 4A). However, within cDCs, a remarkable increase of cDC1 and a decrease of cDC2 subsets were observed in d7 TBI mice (Fig. 4A). Interestingly, expression levels of MHC-CII were increased in total cDCs of blood from d7 TBI mice and further analysis indicated that this increase was specific to the cDC1, but not to the cDC2, compartment (Fig. 4B). Next, we investigated the frequencies of DCs in the skin draining/peripheral lymph nodes (pLN). Analysis indicated normal frequencies of total cDCs and pDCs in the pLNs from d7 TBI mice (Fig. 4C). However, determination of cDC subsets revealed a remarkable decrease of cDC1 and increase of cDC2 subsets in the pLNs of d7 TBI mice (Fig. 4C). Interestingly, immunophenotyping studies on pLN DCs from d7 TBI mice indicated; a striking decrease of MHC- CII expression in all DC subsets, including total cDC, cDC1, cDC2 and pDC subsets (Fig. 4D, left); normal expression levels of CD80 in total cDCs and pDCs. However, within cDCs, CD80 was decreased in cDC1 and increased in cDC2 subsets (Fig. 4D, middle); and CD86 expression was remarkably reduced in total cDC, cDC1 and pDC subsets, even though CD86 expression was normal in cDC2 subset (Fig. 4D, right). Finally, we analyzed the DC compartments in the mesenteric lymph nodes (mLNs) and the data indicated that the frequencies of total cDCs were decreased in d7 TBI mice (Fig. 4E). However, within cDCs, there was a relative increase of cDC1 subset and a decrease in cDC2 subset in the mLNs of d7 TBI mice (Fig. 4E). Immunophenotyping studies on mLN cDCs from d7 TBI mice suggested; normal expression levels of MHC-CII in total cDC, cDC1 and cDC2 subsets (Fig. 4F, left); increased expression levels of CD80 expression in cDC, cDC1 and cDC2 subsets (Fig. 4F, middle); and remarkable upregulation of CD86 in cDC, cDC1 and cDC2 subsets (Fig. 4F, right). Taken together these data demonstrate that TBI affects distribution and functions of specific DC subsets of both circulatory and lymphatic systems.

### TBI affects distribution of DC subsets within solid organs

**Fig. 5:**
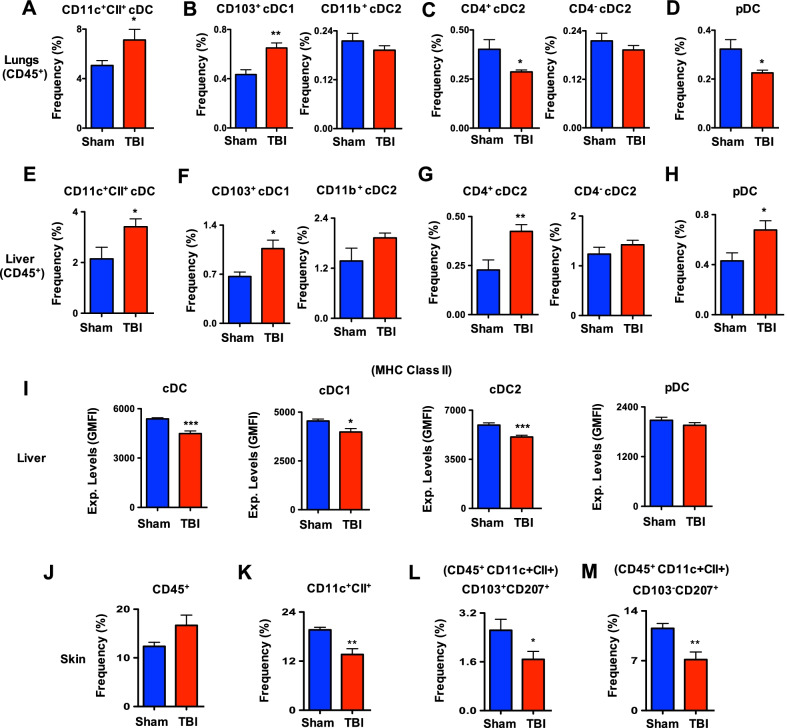
TBI affects DC distribution in solid organs. **A** Frequencies of CD11c^+^CII^high^ cDCs within pre-gated CD45^+^ hematopoietic fraction of lungs from Sham (*n* = 4) and d7-TBI (*n* = 5) mice. Data are representative of two independent experiments. **B** Frequencies of CD103^+^ cDC1 and CD11b^+^ cDC2 subsets within CD45^+^ CD11c^+^CII^high^ cDCs of lungs from Sham (*n* = 4) and d7-TBI (*n* = 5) mice. Data are representative of two independent experiments. **C** Frequencies of CD4^+^ cDC2 and CD4^−^ cDC2 subsets within CD45^+^ CD11c^+^CII^high^ CD103^−^CD11b^+^ cDC2 cells of lungs from Sham (*n* = 4) and d7-TBI (*n* = 5) mice. Data are representative of two independent experiments. **D** Frequencies of CD11c^int^PDCA1^+^ pDCs within pre-gated CD45^+^ hematopoietic fraction of lungs from Sham (*n* = 4) and d7-TBI (*n* = 5) mice. Data are representative of two independent experiments. **E** Frequencies of CD11c^+^CII^high^ cDCs within pre-gated CD45^+^ hematopoietic fraction of liver from Sham (*n* = 4) and d7-TBI (*n* = 5) mice. Data are representative of two independent experiments. **F** Frequencies of CD103^+^ cDC1 and CD11b^+^ cDC2 subsets within CD45^+^ CD11c^+^CII^high^ cDCs of liver from Sham (*n* = 4) and d7-TBI (*n* = 5) mice. Data are representative of two independent experiments. **G** Frequencies of CD4^+^ cDC2 and CD4^−^ cDC2 subsets within CD45^+^ CD11c^+^CII^high^ CD103^−^CD11b^+^ cDC2 cells of liver from Sham (*n* = 4) and d7-TBI (*n* = 5) mice. Data are representative of two independent experiments. **H** Frequencies of CD11c^int^PDCA1^+^ pDCs within pre-gated CD45^+^ hematopoietic fraction of liver from Sham (*n* = 4) and d7-TBI (*n* = 5) mice. Data are representative of two independent experiments. **I** Surface expression levels of MHC-CII in total cDC, cDC1, cDC2 and pDC subsets of liver from Sham (*n* = 4) and d7-TBI (*n* = 5) mice. Shown were GMFI. Data are representative of two independent experiments. **J** Frequencies of CD45^+^ hematopoietic cells from skin of Sham (*n* = 4) and d7-TBI (*n* = 5) mice. Data are representative of two independent experiments. **K** Frequencies of CD11c^+^CII^high^ cDCs within pre-gated CD45^+^ hematopoietic fraction of skin from Sham (*n* = 4) and d7-TBI (*n* = 5) mice. Data are representative of two independent experiments. **L** Frequencies of CD103^+^CD207^+^ dermal DCs within CD45^+^CD11c^+^CII^high^ cells of skin from Sham (*n* = 4) and d7-TBI (*n* = 5) mice. Data are representative of two independent experiments. **M** Frequencies of CD103^−^CD207^+^ Langerhans Cells within CD45^+^CD11c^+^CII^high^ cells of skin from Sham (*n* = 4) and d7-TBI (*n* = 5) mice. Data are representative of two independent experiments. All data represent mean ± SEM. Two-tailed student’s *t* tests were used to assess statistical significance (*P < 0.05, **P < 0.01, ***P < 0.001, ****P < 0.0001)

DC subsets of the solid organs, including lungs, liver and skin, participate in active immune surveillance and maintaining a proper balance of cDC1, cDC2 and pDC subsets is vital for their functions [11, 17, 28, 29]. Emerging studies demonstrate that both non-lymphoid and lymphoid tissue DCs derive from the same precursors in the BM [11] and follow a related differentiation program [30, 31]. To test if TBI impacts the DC composition of solid organs, we determined the frequencies of DC compartments of the lungs. Frequencies of total CD11c^+^CII^+^ cDCs were increased within the CD45^+^ hematopoietic fraction of lungs from d7 TBI mice (Fig. 5A). However, within CD45^+^CD11c^+^CII^+^ cDCs, frequencies of CD103^+^ cDC1 subset were increased and CD11b^+^ cDC2 subset was normal in the lungs of d7 TBI mice (Fig. 5B). Further analysis of CD45^+^CD11c^+^CII^+^CD11b^+^ cDC2 fraction revealed a decrease of CD4^+^ cDC2 subset and normal frequencies of CD4^−^ cDC2 in the lungs of d7 TBI mice (Fig. 5C). Frequencies of CD45^+^CD11c^+^CII^+^ PDCA1^+^ pDCs were reduced in the lungs of d7 TBI mice (Fig. 5D).

Next, we analyzed the DCs of liver from d7 TBI mice. Frequencies of CD11c^+^CII^+^ cDCs within CD45^+^ fraction were elevated in the liver of d7 TBI mice (Fig. 5E). However, within CD45^+^CD11c^+^CII^+^ cDCs, frequencies of CD103^+^ cDC1 subset were increased and CD11b^+^ cDC2 subset was normal in the liver of d7 TBI mice (Fig. 5F). Further analysis of CD45^+^CD11c^+^CII^+^CD11b^+^ cDC2 fraction revealed an increase of CD4^+^ cDC2 subset and normal frequencies of CD4^−^ cDC2 in the liver of d7 TBI mice (Fig. 5G). Frequencies of CD45^+^CD11c^+^CII^+^ PDCA1^+^ pDCs were elevated in the liver of d7 TBI mice (Fig. 5H). Immunophenotyping studies indicated reduced expression levels of MHC-CII in total cDC, cDC1 and cDC2 subsets, whereas normal expression levels of MHC-CII in pDCs from the liver of d7 TBI mice (Fig. 5I).

Finally, we determined the frequencies of DCs in the skin. Frequencies of total CD45^+^ hematopoietic cells were increased in the skin of d7 TBI mice (Fig. 5J). However, within CD45^+^ fraction, the frequencies of CD11c^+^CII^+^ DCs were reduced in the skin of d7 TBI mice (Fig. 5K). Determination of CD11c^+^CII^+^ CD103^+^CD207^+^ dermal DCs and CD11c^+^CII^+^ CD103^−^CD207^+^ Langerhans Cells (LCs) indicated a reduction of both fractions in the skin of d7 TBI mice (Fig. 5L, M). Overall, these data indicate that TBI impacts the distribution and composition of DC subsets within the solid organs that participate in immune-surveillance functions.

### TBI affects early differentiation pathways of DCs in the BM

**Fig. 6:**
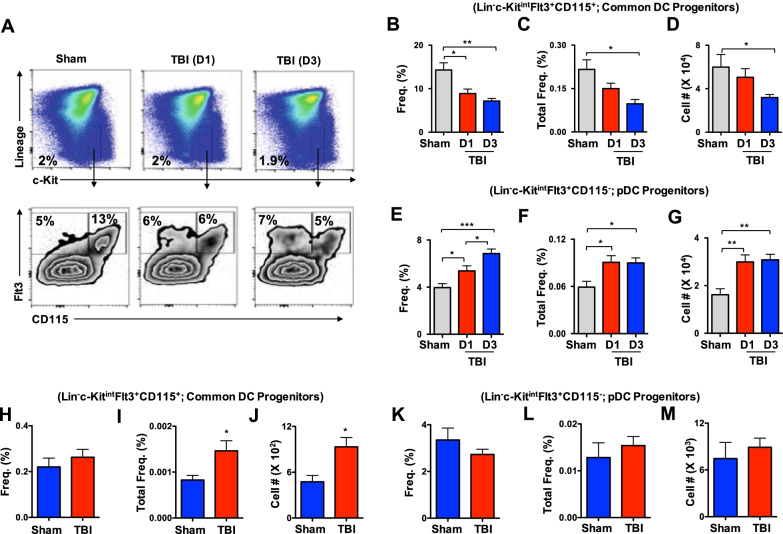
TBI affects early differentiation of DC progenitors in the BM. **A** FACS plots indicating frequencies of Lin^−^c-Kit^+^ Flt3^+^CD115^+^ CDPs and Lin^−^c-Kit^+^ Flt3^+^CD115^−^ pDC progenitors in the BM of Sham (*n* = 4), d1-TBI (*n* = 5) and d3-TBI (*n* = 5) mice. Data are representative of two independent experiments. **B**–**D** Relative frequencies (**B**), overall frequencies (**C**) and absolute numbers (**D**) of Lin^−^c-Kit^+^ Flt3^+^CD115^+^ CDPs in the BM of Sham (*n* = 4), d1-TBI (*n* = 5) and d3-TBI (*n* = 5) mice. Data are representative of two independent experiments. **E**–**G** Relative frequencies (**E**), overall frequencies (**F**) and absolute numbers (**G**) of Lin^−^c-Kit^+^ Flt3^+^CD115^−^ pDC progenitors in the BM of Sham (*n* = 4), d1-TBI (*n* = 5) and d3-TBI (*n* = 5) mice. Data are representative of two independent experiments. **H**–**J** Relative frequencies (**H**), overall frequencies (**I**) and absolute numbers (**J**) of CDPs in the BM of Sham (*n* = 4) and d7-TBI (*n* = 5) mice. Data are representative of two independent experiments. **K**–**M** Relative frequencies (**K**), overall frequencies (**L**) and absolute numbers (**M**) of pDC progenitors in the BM of Sham (*n* = 4) and d7-TBI (*n* = 5) mice. Data are representative of two independent experiments. All data represent mean ± SEM. Mann–Whitney non-parametric tests and two-tailed student’s *t* tests were used to assess statistical significance (*P < 0.05, **P < 0.01, ***P < 0.001, ****P < 0.0001)

Early stages of DC differentiation occur in the BM through commitment of hematopoietic stem cells (HSCs) to common dendritic cell progenitors (CDPs) [32]. Indeed, changes in the frequencies and numbers of CDPs result in abnormal composition of DCs in both lymphoid and non-lymphoid organs [11, 16, 17]. In view of the fact that TBI affects distribution of DCs in almost all organs, we investigated if TBI has an impact on the early stages of DC differentiation. Flow cytometric analyses identified reduced relative frequencies of Lin^−^c-Kit^int^Flt3^+^CD115^+^ CDPs in the BM of d1 and d3 TBI mice (Fig. 6A, B), decreased overall frequencies of CDPs in the BM of d3 TBI mice (Fig. 6A,C), and diminished absolute numbers of CDPs in the BM of d3 TBI mice (Fig. 6A, D). On the other hand, relative frequencies (Fig. 6A, E), overall frequencies (Fig. 6A, F), and absolute numbers (Fig. 6A, G) of Lin^−^c-Kit^int^Flt3^+^CD115^−^ pDC-committed-progenitors were augmented in the BM of both d1 and d3 TBI mice. To study if these changes in the DC progenitors of the BM are consistent at the sub-chronic phase of TBI, we analyzed the BM of d7 TBI mice. Interestingly, our data on CDPs indicated that the relative frequencies were normal (Fig. 6H), overall frequencies were increased (Fig. 6I), and the absolute numbers (Fig. 6J) were augmented in the BM of d7 TBI mice. However, the relative frequencies (Fig. 6K), overall frequencies (Fig. 6L), and absolute numbers (Fig. 6M) remain normal in the BM of d7 TBI mice. These data demonstrate that TBI affects early differentiation program of DCs, especially at the stages of commitment to CDPs.

### TBI suppresses reactive oxygen species production in DC-subsets and progenitors

**Fig. 7:**
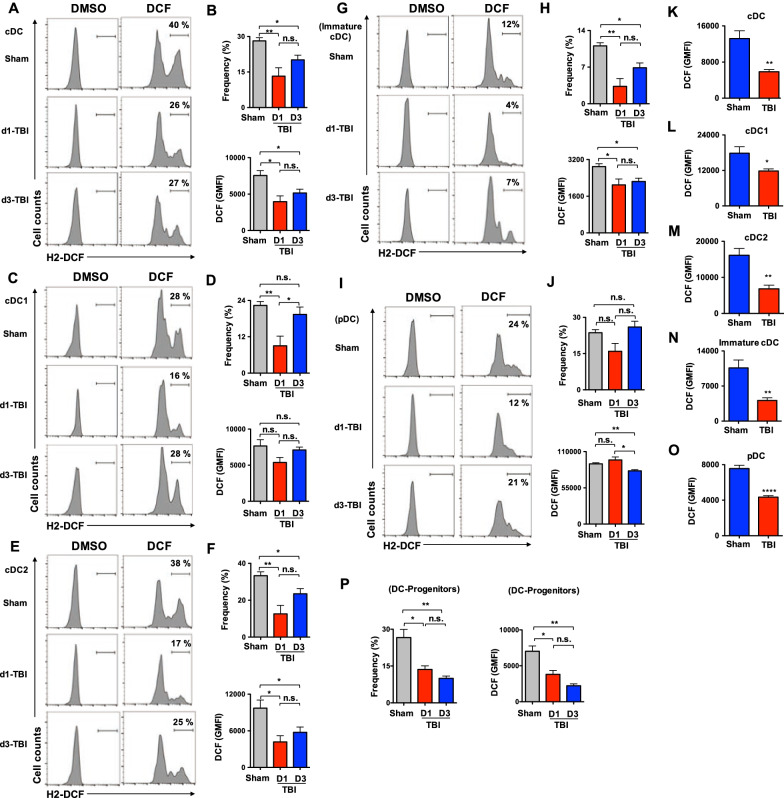
TBI results in diminished levels of ROS in DC subsets and Progenitors. **A** Histograms indicating intracellular ROS levels in CD11c^+^CII^+^ splenic cDCs from sham (*n* = 4), d1-TBI (*n* = 5) and d3-TBI (*n* = 5) mice. Shown were the frequencies of ROS^high^ cells under the indicated gates. Cells treated with DMSO served as negative controls. **B** Frequencies of gated ROS^high^ CD11c^+^CII^+^ splenic cDCs (top) and total intracellular ROS levels (GMFI) in CD11c^+^CII^+^ splenic cDCs (bottom) from sham (*n* = 4), d1-TBI (*n* = 5) and d3-TBI (*n* = 5) mice. **C** Histograms indicating intracellular ROS levels in CD8^+^CD11b^−^CD11c^+^CII^+^ splenic cDC1 subset from sham (*n* = 4), d1-TBI (*n* = 5) and d3-TBI (*n* = 5) mice. Shown were the frequencies of ROS^high^ cells under the indicated gates. Cells treated with DMSO served as negative controls. **D** Frequencies of gated ROS^high^ CD8^+^CD11b^−^CD11c^+^CII^+^ splenic cDC1 subset (top) and total intracellular ROS levels (GMFI) in CD8^+^CD11b^−^CD11c^+^CII^+^ splenic cDC1 subset (bottom) from sham (*n* = 4), d1-TBI (*n* = 5) and d3-TBI (*n* = 5) mice. **E** Histograms indicating intracellular ROS levels in CD8^−^CD11b^+^CD11c^+^CII^+^ splenic cDC2 subset from sham (*n* = 4), d1-TBI (*n* = 5) and d3-TBI (*n* = 5) mice. Shown were the frequencies of ROS^high^ cells under the indicated gates. Cells treated with DMSO served as negative controls. **F** Frequencies of gated ROS^high^ CD8^−^CD11b^+^CD11c^+^CII^+^ splenic cDC2 (top) and total intracellular ROS levels (GMFI) in CD8^−^CD11b^+^CD11c^+^CII^+^ splenic cDC2 (bottom) from sham (*n* = 4), d1-TBI (*n* = 5) and d3-TBI (*n* = 5) mice. **G** Histograms indicating intracellular ROS levels in CD8^−^CD11b^−^CD11c^+^CII^+^ splenic immature cDCs from sham (*n* = 4), d1-TBI (*n* = 5) and d3-TBI (*n* = 5) mice. Shown were the frequencies of ROS^high^ cells under the indicated gates. Cells treated with DMSO served as negative controls. **H** Frequencies of gated ROS^high^ CD8^−^CD11b^−^CD11c^+^CII^+^ splenic immature cDCs (top) and total intracellular ROS levels (GMFI) in CD8^−^CD11b^−^CD11c^+^CII^+^ splenic immature cDCs (bottom) from sham (*n* = 4), d1-TBI (*n* = 5) and d3-TBI (*n* = 5) mice. **I** Histograms indicating intracellular ROS levels in PDCA1^+^CD11c^+^ splenic pDCs from sham (*n* = 4), d1-TBI (*n* = 5) and d3-TBI (*n* = 5) mice. Shown were the frequencies of ROS^high^ cells under the indicated gates. Cells treated with DMSO served as negative controls. **J** Frequencies of gated ROS^high^ PDCA1^+^CD11c^+^ splenic pDCs (top) and total intracellular ROS levels (GMFI) in PDCA1^+^CD11c^+^ splenic pDCs (bottom) from sham (*n* = 4), d1-TBI (*n* = 5) and d3-TBI (*n* = 5) mice. **K**–**O** Histograms indicating intracellular ROS levels (GMFI) in total cDCs (**K**), cDC1 (**L**), cDC2 (**M**), immature cDCs (**N**) and pDCs (**O**) of the spleen from sham (*n* = 5) and d7-TBI (*n* = 5) mice. **P** Frequencies of gated ROS^high^ Lin^−^c-Kit^+^ Flt3^+^CD115^+^ CDPs (left) and total intracellular ROS levels (GMFI) in Lin^−^c-Kit^+^ Flt3^+^CD115^+^ CDPs (right) of the BM of Sham (*n* = 4), d1-TBI (*n* = 5) and d3-TBI (*n* = 5) mice. Data are representative of two independent experiments. All data represent mean ± SEM. Mann–Whitney non-parametric tests and two-tailed student’s *t* tests were used to assess statistical significance (*P < 0.05, **P < 0.01, ***P < 0.001, ****P < 0.0001)

One of the hallmark features of secondary injury following TBI is augmented levels of ROS and oxidative stress in innate immune cells, including microglia and macrophages [33, 34]. Of note, ROS have been shown to play critical and decisive roles at the early differentiation stages of human and mouse DCs in the BM [35–39]. In an attempt to understand the molecular mechanisms that could contribute to altered DC differentiation at early and sub-chronic phases of TBI, we determined the expression levels of ROS in DC subsets and progenitors. Intriguingly, our analysis indicated reduced frequencies of ROS^high^ and total intracellular ROS levels in cDCs of spleen on d1 and d3 after TBI (Fig. 7A, B). Further analysis of cDC subsets revealed a reduction in the frequency of ROS^high^ splenic cDC1 subset from d1 TBI mice (Fig. 7C, D), and a decrease in both frequencies of ROS^high^ and total intracellular ROS levels in splenic cDC2 subset from d1 and d3 TBI mice (Fig. 7E, F). Consistently, analysis of CD8^−^CD11b^−^ immature cDCs exhibited reduced frequencies of ROS^high^ cells and total intracellular ROS levels from d1 and d3 TBI mice (Fig. 7G, H). On the other hand, ROS levels in pDCs indicated normal frequencies of ROS^high^ pDCs from d1 and d3 TBI mice and a reduction in total intracellular ROS levels in pDCs from d3 TBI mice (Fig. 7I, J). To assess the alterations in ROS levels in DC subsets at sub-chronic phase of TBI, we studied DCs from d7 TBI mice. Our data concluded that the total intracellular ROS levels were remarkably reduced in total cDC (Fig. 7K), cDC1 (Fig. 7L), cDC2 (Fig. 7M), immature cDC (Fig. 7N) and pDC (Fig. 7O) subsets from the spleen of d7 TBI mice. Finally, we determined if ROS levels are altered at the early development stages of DCs following TBI. Analysis indicated that both frequencies of ROS^high^ CDPs and total intracellular ROS levels in CDPs were reduced in the BM of d1 and d3 TBI mice (Fig. 7P). In essence, these data established that the intracellular ROS levels were reduced in DC progenitors and differentiated DCs at both early and sub-chronic phases of TBI.

## Discussion

Mounting evidence established that TBI affects organs outside the CNS. Mirzayan et al. reported that TBI causes augmented migration and infiltration of immune cells to peripheral organs, such as lungs and liver, which eventually led to histopathological changes and various degrees of organ dysfunction [40]. Interestingly, their study concluded that the composition of spleen and kidney were not altered in response to TBI. On the other hand, a series of studies established that peripheral immune cells play a critical role in the overall pathology after brain injury. Indeed, splenectomy in rats immediately after TBI resulted in decreased expression of pro-inflammatory cytokines, mortality rate, improved cognitive function [41] and attenuated neurodegeneration and CCL20 chemokine expression in the brain [18]. Most of the peripheral innate immune cells, including neutrophils, monocytes and macrophages, are believed to respond at the early phase and decline within few days after TBI [42]. Intriguingly, our present data suggest that peripheral DC composition and numbers are altered within 24 h of TBI and exhibited sustained changes even after 7 days of TBI. However, it is unclear, at present, if peripheral DCs migrate to the injured site in the brain and participate in inflammation and neurodegeneration. Future studies with an in-depth focus on identifying the involvement of DCs in neuroinflammation and pathophysiology of CNS would be essential.

Emerging studies have unequivocally established the contribution of oxidative stress to secondary injury in TBI pathology, including development of cerebral edema, inflammation, and the secondary neuronal damage found post-TBI. Disrupted blood flow after TBI leads to cerebral hypoxia or ischemia with the consequent decrease of oxygen and glucose supply to the CNS [43]. In addition, mitochondrial dysfunction caused by TBI results in marked accumulation of ROS in the brain [44]. In sharp contrast, our studies identified that ROS levels were rather diminished in both differentiated DCs and DC subsets at acute and sub-chronic phases of TBI. In keeping with our findings, neutrophil ROS production in patients with moderate or severe TBI was significantly lower than that of healthy age- and sex-matched individuals [42]. Given the importance of ROS in the differentiation and functional integrity of DCs [35–39], we speculate that alterations in the intracellular levels of ROS might be, at least partially, responsible for DC alterations in peripheral organs after TBI. Furthermore, TBI results in elevated systemic levels of proinflammatory cytokines including TNFα, IL1β and IL6 [45–48]. We [24–27, 49], as well as others, have demonstrated that exaggerated systemic levels of inflammatory cytokines, such as TNFα, IL1β, IFNα, IFNγ, and IL6, affects early differentiation of immune cells [50–52]. Of note, increased systemic levels of proinflammatory cytokines suppress DC differentiation program of HSCs within the BM. Therefore, based on the current knowledge, we believe that the DC differentiation defects after TBI are caused by deregulated ROS levels and/or augmented signaling by proinflammatory cytokines in HSCs. Further studies will be needed to establish the precise mechanisms through which TBI alters DC development and functions.

DCs are regarded as the most professional antigen presenting cells and indispensable in initiating T-, B- and NK-cell-mediated adaptive immune responses against viral, fungal and bacterial infections [9–12]. In particular, DCs are essential to provide immunity against lung infections, including viral and bacterial pneumonia [28, 53–57]. A recent study reported rapid maturation of DCs, possibly due to increased phosphorylation of Flt3 receptor, metabolic activity, protein synthesis, lysosomal activity, and inflammatory properties within few hours of TBI [58]. On the other hand, data from this current study document that TBI directly affects the pool and composition of DCs in almost all lymphoid and non-lymphoid organs, including lungs at early and sub-chronic phases of TBI. These altered frequencies and numbers of cDC1, cDC2 and pDCs subsets in each tissue may be, at least in part, responsible for the immune dysfunction caused by TBI. More importantly, our analyses concluded that TBI affects the immunogenicity of DCs, such as altered surface expression of MHC CI and CII proteins and co-stimulatory molecules—CD80 and CD86, which may eventually lead to defective functions of DCs.

Infections are a leading cause of morbidity and mortality in patients with acute CNS injury, such as TBI, stroke and spinal cord injury [19–21, 59]. Indeed, there exists a direct correlation between infections and poorer clinical outcomes overall [60]. More importantly, infections were found to be the key driver of ~ 19% of mortality in TBI patients, that survived at least 1 year after TBI [61]. Mounting evidences indicate that nosocomial infections (following TBI) are major contributors of not only to mortality, but also inducers of deleterious long-term consequences in patients [62]. Furthermore, infections acquired after TBI may trigger pathophysiological sequelae and exacerbate neurodegeneration [63, 64], intracranial hypertension and an associated need for longer duration on mechanical ventilation [65–69]. Indeed, survivors of brain trauma are 2.3–4.3 times more likely to die than general population, with increased risk of death from pneumonia or sepsis [61]. Recent studies indicated that up to 75% of severe TBI patients have been reported to develop sepsis, that itself is linked to high rates of morbidity, cognitive impairment, anxiety and depression, mediated multi-organ failure [70]. Post-traumatic immune insults are life-threatening complications in patients with moderate–severe TBI and observed in up to 55% of patients [70]. The most common nosocomial infections in TBI patients include, pneumonia/lower respiratory tract infections, urinary tract infections and surgical site infections [19, 20, 70–72].

While the detrimental consequences of viral and bacterial infections in TBI patients have been unequivocally established, pathophysiological mechanisms responsible for high susceptibility to immune insults and nosocomial infections in TBI patients remain unclear. Data from the current study, for the first time, identified that DC-development and functions are diminished after TBI. Based on the functional significance of DCs in initiating B and T cell responses against infections, it is tempting to speculate that reduced DC-mediated immunity might be responsible for immunodeficiencies observed in TBI patients. More importantly, our studies demonstrate that TBI causes a differential impact among DC (cDC1 vs. cDC2 vs. pDC) subsets. Given the functional diversities and physiological relevance of cDC1, cDC2 and pDC subsets in generating immunity against bacterial, viral, and fungal infections, it would be of major clinical significance to study DC physiology in TBI patients, especially in individuals with immune insults. A clearer understanding on the precise mechanisms through which TBI suppress DCs would be helpful in restoring DC functions in TBI patients. Our studies would be useful in designing DC-based novel therapeutic strategies toward treatment of patients suffering from TBI.

## Conclusions

Our data demonstrate, for the first time, that TBI affects the distribution pattern of DCs and induces an imbalance among DC subsets in both lymphoid and non-lymphoid organs. In addition, the current study demonstrates that TBI results in reduced levels of ROS in DCs at both acute and sub-chronic phases of TBI, which may explain altered DC differentiation paradigm following TBI. A deeper understanding on the molecular mechanisms that contribute to DC defects following TBI would be essential and beneficial in treating infections in patients with acute CNS injuries, such as TBI, stroke and spinal cord injury.

## Data Availability

The data that support the findings of this study are available from corresponding author on reasonable request.

## Abbreviations

TBI: Traumatic brain injury
DC: Dendritic cells
CDP: Common dendritic cell progenitor
pDC-P: Plasmacytoid DC progenitor
cDCs: Conventional or classical DCs
pDCs: Plasmacytoid DCs
CNS: Central nervous system
ROS: Reactive oxygen species
BM: Bone marrow
pLN: Peripheral lymph node
mLN: Peripheral lymph node

## References

[1] Jassam YN, Izzy S, Whalen M, McGavern DB, El Khoury J (2017). Neuroimmunology of traumatic brain injury: time for a paradigm shift. Neuron.

[2] Maas AI, Stocchetti N, Bullock R (2008). Moderate and severe traumatic brain injury in adults. Lancet Neurol.

[3] Langlois JA, Rutland-Brown W, Wald MM (2006). The epidemiology and impact of traumatic brain injury: a brief overview. J Head Trauma Rehabil.

[4] Nguyen R, Fiest KM, McChesney J, Kwon CS, Jette N, Frolkis AD, Atta C, Mah S, Dhaliwal H, Reid A (2016). The international incidence of traumatic brain injury: a systematic review and meta-analysis. Can J Neurol Sci.

[5] Roozenbeek B, Maas AI, Menon DK (2013). Changing patterns in the epidemiology of traumatic brain injury. Nat Rev Neurol.

[6] Skolnick BE, Maas AI, Narayan RK, van der Hoop RG, MacAllister T, Ward JD, Nelson NR, Stocchetti N, Investigators ST (2014). A clinical trial of progesterone for severe traumatic brain injury. N Engl J Med.

[7] Bramlett HM, Dietrich WD (2015). Long-term consequences of traumatic brain injury: current status of potential mechanisms of injury and neurological outcomes. J Neurotrauma.

[8] Ganguly D, Haak S, Sisirak V, Reizis B (2013). The role of dendritic cells in autoimmunity. Nat Rev Immunol.

[9] Steinman RM (2012). Decisions about dendritic cells: past, present, and future. Annu Rev Immunol.

[10] Belz GT, Nutt SL (2012). Transcriptional programming of the dendritic cell network. Nat Rev Immunol.

[11] Merad M, Sathe P, Helft J, Miller J, Mortha A (2013). The dendritic cell lineage: ontogeny and function of dendritic cells and their subsets in the steady state and the inflamed setting. Annu Rev Immunol.

[12] Miller JC, Brown BD, Shay T, Gautier EL, Jojic V, Cohain A, Pandey G, Leboeuf M, Elpek KG, Helft J (2012). Deciphering the transcriptional network of the dendritic cell lineage. Nat Immunol.

[13] Bigley V, Haniffa M, Doulatov S, Wang XN, Dickinson R, McGovern N, Jardine L, Pagan S, Dimmick I, Chua I (2011). The human syndrome of dendritic cell, monocyte, B and NK lymphoid deficiency. J Exp Med.

[14] Dickinson RE, Griffin H, Bigley V, Reynard LN, Hussain R, Haniffa M, Lakey JH, Rahman T, Wang XN, McGovern N (2011). Exome sequencing identifies GATA-2 mutation as the cause of dendritic cell, monocyte, B and NK lymphoid deficiency. Blood.

[15] Hambleton S, Salem S, Bustamante J, Bigley V, Boisson-Dupuis S, Azevedo J, Fortin A, Haniffa M, Ceron-Gutierrez L, Bacon CM (2011). IRF8 mutations and human dendritic-cell immunodeficiency. N Engl J Med.

[16] Mildner A, Jung S (2014). Development and function of dendritic cell subsets. Immunity.

[17] Sichien D, Lambrecht BN, Guilliams M, Scott CL (2017). Development of conventional dendritic cells: from common bone marrow progenitors to multiple subsets in peripheral tissues. Mucosal Immunol.

[18] Das M, Leonardo CC, Rangooni S, Pennypacker KR, Mohapatra S, Mohapatra SS (2011). Lateral fluid percussion injury of the brain induces CCL20 inflammatory chemokine expression in rats. J Neuroinflamm.

[19] Faden AI, Barrett JP, Stoica BA, Henry RJ (2021). Bidirectional brain-systemic interactions and outcomes after TBI. Trends Neurosci.

[20] Meisel C, Schwab JM, Prass K, Meisel A, Dirnagl U (2005). Central nervous system injury-induced immune deficiency syndrome. Nat Rev Neurosci.

[21] Sharma R, Shultz SR, Robinson MJ, Belli A, Hibbs ML, O'Brien TJ, Semple BD (2019). Infections after a traumatic brain injury: the complex interplay between the immune and neurological systems. Brain Behav Immun.

[22] Israelsson C, Kylberg A, Bengtsson H, Hillered L, Ebendal T (2014). Interacting chemokine signals regulate dendritic cells in acute brain injury. PLoS ONE.

[23] Gallizioli M, Miro-Mur F, Otxoa-de-Amezaga A, Cugota R, Salas-Perdomo A, Justicia C, Brait VH, Ruiz-Jaen F, Arbaizar-Rovirosa M, Pedragosa J (2020). Dendritic cells and microglia have non-redundant functions in the inflamed brain with protective effects of type 1 cDCs. Cell Rep.

[24] Nakagawa MM, Chen H, Rathinam CV (2018). Constitutive activation of NF-kappaB pathway in hematopoietic stem cells causes loss of quiescence and deregulated transcription factor networks. Front Cell Dev Biol.

[25] Nakagawa MM, Davis H, Rathinam CV (2018). A20 deficiency in multipotent progenitors perturbs quiescence of hematopoietic stem cells. Stem Cell Res.

[26] Nakagawa MM, Rathinam CV (2018). Constitutive activation of the canonical NF-kappaB pathway leads to bone marrow failure and induction of erythroid signature in hematopoietic stem cells. Cell Rep.

[27] Nakagawa MM, Thummar K, Mandelbaum J, Pasqualucci L, Rathinam CV (2015). Lack of the ubiquitin-editing enzyme A20 results in loss of hematopoietic stem cell quiescence. J Exp Med.

[28] Eddy WE, Gong KQ, Bell B, Parks WC, Ziegler SF, Manicone AM (2017). Stat5 is required for CD103(+) dendritic cell and alveolar macrophage development and protection from lung injury. J Immunol.

[29] Guilliams M, Lambrecht BN, Hammad H (2013). Division of labor between lung dendritic cells and macrophages in the defense against pulmonary infections. Mucosal Immunol.

[30] Ginhoux F, Liu K, Helft J, Bogunovic M, Greter M, Hashimoto D, Price J, Yin N, Bromberg J, Lira SA (2009). The origin and development of nonlymphoid tissue CD103+ DCs. J Exp Med.

[31] Helft J, Ginhoux F, Bogunovic M, Merad M (2010). Origin and functional heterogeneity of non-lymphoid tissue dendritic cells in mice. Immunol Rev.

[32] Onai N, Obata-Onai A, Schmid MA, Ohteki T, Jarrossay D, Manz MG (2007). Identification of clonogenic common Flt3+M-CSFR+ plasmacytoid and conventional dendritic cell progenitors in mouse bone marrow. Nat Immunol.

[33] Hall ED, Wang JA, Miller DM (2012). Relationship of nitric oxide synthase induction to peroxynitrite-mediated oxidative damage during the first week after experimental traumatic brain injury. Exp Neurol.

[34] Kumar A, Barrett JP, Alvarez-Croda DM, Stoica BA, Faden AI, Loane DJ (2016). NOX2 drives M1-like microglial/macrophage activation and neurodegeneration following experimental traumatic brain injury. Brain Behav Immun.

[35] Del Prete A, Zaccagnino P, Di Paola M, Saltarella M, Oliveros Celis C, Nico B, Santoro G, Lorusso M (2008). Role of mitochondria and reactive oxygen species in dendritic cell differentiation and functions. Free Radic Biol Med.

[36] Sattler M, Winkler T, Verma S, Byrne CH, Shrikhande G, Salgia R, Griffin JD (1999). Hematopoietic growth factors signal through the formation of reactive oxygen species. Blood.

[37] Sheng KC, Pietersz GA, Tang CK, Ramsland PA, Apostolopoulos V (2010). Reactive oxygen species level defines two functionally distinctive stages of inflammatory dendritic cell development from mouse bone marrow. J Immunol.

[38] Sinha A, Singh A, Satchidanandam V, Natarajan K (2006). Impaired generation of reactive oxygen species during differentiation of dendritic cells (DCs) by *Mycobacterium tuberculosis* secretory antigen (MTSA) and subsequent activation of MTSA-DCs by mycobacteria results in increased intracellular survival. J Immunol.

[39] Zaccagnino P, Saltarella M, Maiorano S, Gaballo A, Santoro G, Nico B, Lorusso M, Del Prete A (2012). An active mitochondrial biogenesis occurs during dendritic cell differentiation. Int J Biochem Cell Biol.

[40] Mirzayan MJ, Probst C, Krettek C, Samii M, Pape HC, van Griensven M, Samii A (2008). Systemic effects of isolated brain injury: an experimental animal study. Neurol Res.

[41] Li M, Li F, Luo C, Shan Y, Zhang L, Qian Z, Zhu G, Lin J, Feng H (2011). Immediate splenectomy decreases mortality and improves cognitive function of rats after severe traumatic brain injury. J Trauma.

[42] Alam A, Thelin EP, Tajsic T, Khan DZ, Khellaf A, Patani R, Helmy A (2020). Cellular infiltration in traumatic brain injury. J Neuroinflamm.

[43] Khatri N, Thakur M, Pareek V, Kumar S, Sharma S, Datusalia AK (2018). Oxidative stress: major threat in traumatic brain injury. CNS Neurol Disord Drug Targets.

[44] Angeloni C, Prata C, Dalla Sega FV, Piperno R, Hrelia S (2015). Traumatic brain injury and NADPH oxidase: a deep relationship. Oxid Med Cell Longev.

[45] Keel M, Trentz O (2005). Pathophysiology of polytrauma. Injury.

[46] Lu J, Goh SJ, Tng PY, Deng YY, Ling EA, Moochhala S (2009). Systemic inflammatory response following acute traumatic brain injury. Front Biosci (Landmark Ed).

[47] Weaver LC, Bao F, Dekaban GA, Hryciw T, Shultz SR, Cain DP, Brown A (2015). CD11d integrin blockade reduces the systemic inflammatory response syndrome after traumatic brain injury in rats. Exp Neurol.

[48] Wilcockson DC, Campbell SJ, Anthony DC, Perry VH (2002). The systemic and local acute phase response following acute brain injury. J Cereb Blood Flow Metab.

[49] Rathinam C (2015). The 'inflammatory' control of hematopoietic stem cells. Oncotarget.

[50] Baldridge MT, King KY, Goodell MA (2011). Inflammatory signals regulate hematopoietic stem cells. Trends Immunol.

[51] King KY, Goodell MA (2011). Inflammatory modulation of HSCs: viewing the HSC as a foundation for the immune response. Nat Rev Immunol.

[52] Mirantes C, Passegue E, Pietras EM (2014). Pro-inflammatory cytokines: emerging players regulating HSC function in normal and diseased hematopoiesis. Exp Cell Res.

[53] Beshara R, Sencio V, Soulard D, Barthelemy A, Fontaine J, Pinteau T, Deruyter L, Ismail MB, Paget C, Sirard JC (2018). Alteration of Flt3-Ligand-dependent de novo generation of conventional dendritic cells during influenza infection contributes to respiratory bacterial superinfection. PLoS Pathog.

[54] GeurtsvanKessel CH, Willart MA, van Rijt LS, Muskens F, Kool M, Baas C, Thielemans K, Bennett C, Clausen BE, Hoogsteden HC (2008). Clearance of influenza virus from the lung depends on migratory langerin+CD11b- but not plasmacytoid dendritic cells. J Exp Med.

[55] Nobs SP, Schneider C, Heer AK, Huotari J, Helenius A, Kopf M (2016). PI3Kgamma is critical for dendritic cell-mediated CD8+ T cell priming and viral clearance during influenza virus infection. PLoS Pathog.

[56] Soto JA, Galvez NMS, Andrade CA, Pacheco GA, Bohmwald K, Berrios RV, Bueno SM, Kalergis AM (2020). The role of dendritic cells during infections caused by highly prevalent viruses. Front Immunol.

[57] Vasilevsky S, Colino J, Puliaev R, Canaday DH, Snapper CM (2008). Macrophages pulsed with *Streptococcus pneumoniae* elicit a T cell-dependent antibody response upon transfer into naive mice. J Immunol.

[58] Zhang J, Li Z, Chandrasekar A, Li S, Ludolph A, Boeckers TM, Huber-Lang M, Roselli F, Olde Heuvel F (2022). Fast maturation of splenic dendritic cells upon TBI is associated with FLT3/FLT3L signaling. Front Immunol.

[59] Iadecola C, Anrather J (2011). The immunology of stroke: from mechanisms to translation. Nat Med.

[60] Kesinger MR, Kumar RG, Wagner AK, Puyana JC, Peitzman AP, Billiar TR, Sperry JL (2015). Hospital-acquired pneumonia is an independent predictor of poor global outcome in severe traumatic brain injury up to 5 years after discharge. J Trauma Acute Care Surg.

[61] Harrison-Felix C, Kolakowsky-Hayner SA, Hammond FM, Wang R, Englander J, Dams-O'Connor K, Kreider SE, Novack TA, Diaz-Arrastia R (2012). Mortality after surviving traumatic brain injury: risks based on age groups. J Head Trauma Rehabil.

[62] Andraweera N, Seemann R (2016). Acute rehospitalisation during the first 3 months of in-patient rehabilitation for traumatic brain injury. Aust Health Rev.

[63] Failli V, Kopp MA, Gericke C, Martus P, Klingbeil S, Brommer B, Laginha I, Chen Y, DeVivo MJ, Dirnagl U, Schwab JM (2012). Functional neurological recovery after spinal cord injury is impaired in patients with infections. Brain.

[64] Perry VH, Newman TA, Cunningham C (2003). The impact of systemic infection on the progression of neurodegenerative disease. Nat Rev Neurosci.

[65] Bronchard R, Albaladejo P, Brezac G, Geffroy A, Seince PF, Morris W, Branger C, Marty J (2004). Early onset pneumonia: risk factors and consequences in head trauma patients. Anesthesiology.

[66] Esnault P, Nguyen C, Bordes J, D'Aranda E, Montcriol A, Contargyris C, Cotte J, Goutorbe P, Joubert C, Dagain A (2017). Early-onset ventilator-associated pneumonia in patients with severe traumatic brain injury: incidence, risk factors, and consequences in cerebral oxygenation and outcome. Neurocrit Care.

[67] Jovanovic B, Milan Z, Djuric O, Markovic-Denic L, Karamarkovic A, Gregoric P, Doklestic K, Avramovic J, Velickovic J, Bumbasirevic V (2016). Twenty-eight-day mortality of blunt traumatic brain injury and co-injuries requiring mechanical ventilation. Med Princ Pract.

[68] Magnotti LJ, Croce MA, Fabian TC (2004). Is ventilator-associated pneumonia in trauma patients an epiphenomenon or a cause of death?. Surg Infect (Larchmt).

[69] Safdar N, Dezfulian C, Collard HR, Saint S (2005). Clinical and economic consequences of ventilator-associated pneumonia: a systematic review. Crit Care Med.

[70] McDonald SJ, Sharkey JM, Sun M, Kaukas LM, Shultz SR, Turner RJ, Leonard AV, Brady RD, Corrigan F (2020). Beyond the brain: peripheral interactions after traumatic brain injury. J Neurotrauma.

[71] Das M, Mohapatra S, Mohapatra SS (2012). New perspectives on central and peripheral immune responses to acute traumatic brain injury. J Neuroinflamm.

[72] Hazeldine J, Lord JM, Belli A (2015). Traumatic brain injury and peripheral immune suppression: primer and prospectus. Front Neurol.

